# How Many Veteran COVID‐19 Cases Were There during the Pandemic?

**DOI:** 10.1002/jmv.71040

**Published:** 2026-06-29

**Authors:** Wathsala N. Widanagamaachchi, Ariana R. Callahan, Tina M. Willson, Bree Barbeau, Julia E. Lewis, Christian D. Dalton, Vanessa W. Stevens, Matthew Samore, Nathorn Chaiyakunapruk, Makoto M. Jones

**Affiliations:** ^1^ Division of Epidemiology, Department of Internal Medicine, Spencer Fox Eccles School of Medicine University of Utah Salt Lake City Utah USA; ^2^ IDEAS Center, Veterans Affairs Salt Lake City Healthcare System Salt Lake City Utah USA; ^3^ Division of Infectious Diseases University of Utah Salt Lake City Utah USA; ^4^ Department of Pharmacotherapy, College of Pharmacy University of Utah Salt Lake City Utah USA; ^5^ Department of Social and Administrative Pharmacy, Faculty of Pharmaceutical Sciences Chulalongkorn University Bangkok Thailand

**Keywords:** bias, COVID‐19, incidence, pandemics, policy, United States, veterans

## Abstract

This study estimates the incidence of symptomatic COVID‐19 cases, both documented and undocumented, among U.S. Veterans across demographic groups from the beginning of the pandemic to the end of the public health emergency on May 11, 2023. By analyzing a cohort of Veterans alive as of March 1, 2020, we extended a mortality‐based estimation approach to measure COVID‐19 incidence. We relaxed the assumptions of a constant infection fatality rate (IFR) over time and across age groups and broadened the model from considering only excess respiratory deaths to including excess all‐cause deaths. Descriptive analyses were performed to understand differential ascertainment biases among demographic groups. Resulting estimates suggested a significantly higher number of COVID‐19 cases among Veterans than those documented in the electronic health record. We also identified varying biases among different demographic groups. These estimates offer a clearer view of COVID‐19's impact on Veterans, accounting for missed cases among those who sought care outside of the VA. Differences between documented and estimated cases were substantial. Policymakers should recognize that actual numbers are likely much higher than documented and that documented rates may not be directly comparable across populations or time periods.

## 1 Introduction

It was challenging to count total COVID‐19 infections when not even all medically‐attended infections were captured in an integrated healthcare system's electronic health records. According to the US Department of Veterans Affairs (VA), there were 761,132 cases of COVID‐19 reported as of June 14, 2023, representing about 8% of enrolled Veterans [1, 2] —a sizeable but likely incomplete number. Understanding the full scope of infection is essential for planning future resource allocation and health services, particularly as the impact is not uniformly distributed across the population. Documented case counts may systematically underrepresent infection burden in certain demographic groups, not merely because of differences in disease manifestation (e.g., among the elderly), but because of structural inequities in testing access and healthcare utilization [3, 4]. If ascertainment bias itself varies by but also correlates with historical inequities, then inequities may be compounded by even egalitarian policy responses. Correcting for differential ascertainment establishes a more accurate baseline for evaluating disparate impact and provides a methodological template that could be applied more rapidly in future health emergencies.

Cases may be missed for many reasons (e.g., healthcare access issues, lack of tests or home testing, or perceptions about the virus). Additionally, the Centers for Disease Control and Prevention (CDC) estimates that approximately 5%–30% of COVID‐19 cases in the US were asymptomatic, meaning that individuals with asymptomatic infections were very likely to go undetected [5]. Therefore, the actual number of Veteran COVID‐19 cases was likely much higher than apparent due to incomplete ascertainment. Asymptomatic cases still infect others and ultimately count towards those with cumulative immunity as pandemics progress, affecting transmission dynamics.

During the pandemic, substantial efforts were put into collecting and integrating multiple, diverse data sources for case counting [6, 7, 8, 9]. This involved combining information from different entities, as well as sources (e.g., surveillance testing, clinical diagnosis, and hospitalizations). However, integrating diverse data feeds often takes time and is limited by availability, compatibility, and quality. Additionally, research has been conducted to allow inferred but potentially more timely estimates based on conventional count data and common pandemic characteristics that are commonly published and updated over time [6]. These advances have enabled researchers to better estimate the number of COVID‐19 cases in the US over the entire pandemic period.

Our work applies and extends these methods to estimate the cumulative incidence of symptomatic COVID‐19 cases within the US Veteran population. To achieve this, we utilize the Mortality Mapping (mMAP), an established epidemiological and statistical analysis approach developed by Lu et al., [6] to analyze data pertaining to Veterans and their exposure to the virus. We provide estimates of COVID‐19 infections among the entire Veteran population in the US as well as across different demographic groups.

## 2 Methods

### 2.1 Data Sources

VA maintains the largest integrated healthcare system in the US, with detailed records of the care it provides [10]. These records include demographic details, healthcare utilization, and service history. Data are stored in VA's Corporate Data Warehouse (CDW). Researchers can access the data via the Veterans Informatics and Computing Infrastructure (VINCI). The CDW also houses the VA COVID‐19 Shared Data Resource [11], a specialized data domain that includes COVID‐19 related information for Veterans, such as laboratory tests, hospitalizations, deaths, vaccinations, and treatments.

### 2.2 Definitions

We considered all Veterans enrolled in the Veterans Health Administration (VHA) who were alive as of March 1, 2020, totaling about 9 M Veterans. Using the VA COVID‐19 Shared Data Resource, we identified documented COVID‐19 cases and deaths among the Veteran population from March 1, 2020 through the end of the public health emergency in the U.S. on May 11, 2023 [12]. A COVID‐19 case was identified by either the first positive COVID‐19 laboratory test (PCR or antigen) conducted at a VA medical center, or through evidence of a positive laboratory test found in clinical notes analyzed by natural language processing (NLP). Any death occurring within 30 days of a COVID‐19 case date was considered a COVID‐19‐associated death.

The mMAP approach incorporates excess respiratory deaths measurements. We calculated the excess respiratory deaths by considering the excess influenza and pneumonia deaths by ICD‐10‐CM [13] within the Veteran population over the same time period. Excess counts were determined by comparing the average yearly counts to the documented counts. During the pandemic, respiratory deaths may not have been medically attended at VA facilities and thus would have no VA‐documented diagnosis codes. As an alternative, we also considered excess all‐cause deaths during the time period as COVID‐19 related and therefore extracted the number of excess all‐cause deaths within the Veteran population over the same time period. This method has the benefit of being complete, albeit lagged by several weeks and not specific, during the pandemic.

The pandemic was divided into six waves by a review of US case data [14] and identifying peaks across time. The waves were defined as follows—Wave 1: March 01 to September 08, 2020, Wave 2: September 09, 2020, to June 19, 2021, Wave 3 (Delta): June 20 to November 26, 2021, Wave 4 (Omicron): November 27, 2021, to March 21, 2022, Wave 5: March 22, to October 12, 2022, and Wave 6: October 13, 2022, to June 14, 2023.

We gathered two key demographic characteristics for the Veteran cohort: race and age. The VA collects self‐reported data on race. Its categories include *American Indian or Alaska Native, Asian*, *Black or African American*, *Native Hawaiian or Other Pacific Islander*, and *White*. Veterans reporting multiple races were classified as *Multiracial* for this analysis. Additionally, an *Unknown* category was created for Veterans with missing or unspecified data.

Age ranges considered for the analysis were *< 20*, *20–29*, *30–39*, *40–49*, *50–59*, *60–69*, *70–79*, and *80+* years, with *Unknown* used for missing age data.

## 3 Methods

Previous work by Lu et al. [6] introduced several complementary methods for estimating early cumulative incidence of symptomatic COVID‐19 cases in the US. Among these, mMAP stands out as a more comprehensive approach that can be applied across the entire pandemic period. Consequently, this paper focuses on utilizing the mMAP method to estimate the spread of COVID‐19 cases within the Veteran population across the pandemic. For context, we provide a concise overview of the mMAP approach and its data sources; for more detailed information, readers are referred to the original works. We also examined two other approaches proposed by Lu et al. [6] namely Divergence and COVID Scaling. For more detailed information on this, readers are referred to the supplementary documents.

mMAP is a time series deconvolution method which infers the true COVID‐19 case counts from documented COVID‐19 deaths. It uses symptom onset to death distribution, smoothed time series of documented COVID‐19 deaths, and symptomatic case fatality rate (sCFR) to estimate the distribution of symptomatic COVID‐19 cases. The sCFR is calculated based on the infection fatality rate (IFR) and asymptomatic rate (AR). Lu et al. present two versions of the mMAP approach: an unadjusted version, which uses the pre‐COVID‐19 baseline information and an adjusted version, which represents the best estimate considering all information available after COVID‐19. The mMAP adjusted version uses excess influenza and pneumonia deaths, assuming that they can be attributable to COVID‐19 during the pandemic, to account for the undocumented COVID‐19 deaths. It also incorporates newer information from serological testing via a lower IFR and AR for COVID‐19. We explored both versions of the mMAP approach; however, for the scope of this paper, we present results using the mMAP adjusted version.

To relax the assumptions of the mMAP adjusted version, the Farrington method [15] was used to extract the excess all‐cause deaths within the Veteran population. The Farrington method is based on a quasi‐Poisson regression model that is commonly used to estimate excess deaths. It was originally proposed by Farrington et al. [15] and extended by Noufaily et al. [16]. The expected baseline all‐cause death rate was estimated directly from the Veteran population using the death index derived from the Farrington method, ensuring that excess deaths were defined relative to a Veteran‐specific baseline. Deaths exceeding the 95% upper bound of this expected count were classified as excess. Additionally, we used the systematic analysis on COVID‐19 infection–fatality ratios (IFRs) presented by Sorensen et al. [17] for computing the age‐adjusted IFR values of each COVID‐19 wave. Finally, we also relaxed the assumption that the IFR was static through the pandemic, allowing this to vary using published estimates by wave.

### 3.1 Analysis

Using the mMAP approach, we estimated incident symptomatic COVID‐19 cases among Veterans across all six waves. The parameter values initially presented by Lu et al. [6] were calculated based on data from the early weeks of the pandemic. To better estimate the number of COVID‐19 cases over the entire pandemic period, we updated IFR values using the latest COVID‐19 case and death counts from CDC [14]. We calculated both a constant IFR value across all COVID‐19 waves and individual IFR values for each wave, which were then utilized in our case estimations. We also computed age‐adjusted IFR values for each wave to provide a more accurate assessment of the COVID‐19 pandemic's impact by taking into account variations in age distribution within the Veteran population.

The mMAP adjusted version by Lu et al. [6] considered excess deaths due to influenza and pneumonia (i.e. respiratory) as potential COVID‐19 deaths. However, as the understanding of the COVID‐19 pandemic's impact evolved, it became clear that COVID‐19 could lead to excess mortality from a variety of causes, not limited to respiratory illnesses. Therefore, we broadened the assumption to include excess all‐cause deaths during the specified time period. Additionally, to provide a more detailed understanding of the pandemic's impact, we analyzed case estimations across Veteran groups identified by two demographic factors (race and age).

## 4 Results

### 4.1 Veteran Population

We identified a total of 9,027,914 Veterans who met our study criteria. The majority of these Veterans were *White* (67.28%), followed by *Black or African American* Veterans at 15.62%. Additionally, 13.26% of the Veterans marked their race as *Unknown*, while the remaining racial groups collectively accounted for less than 5%. The data also revealed that 90.18% of the Veterans were *male*, and 11.98% were aged 80 years or older.

### 4.2 COVID‐19 Cases and Deaths

**Figure 1:**
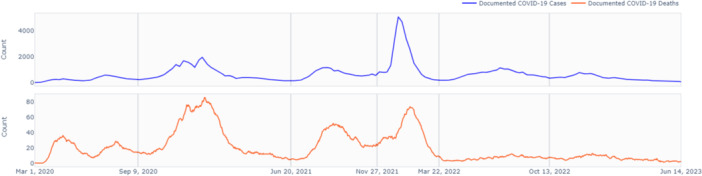
The daily totals of documented COVID‐19 cases (‘Blue’ line) and known COVID‐19‐associated deaths (‘Red’ line) among Veterans during the pandemic.

Over the study period, there were 761,132 documented COVID‐19 cases and 24,366 documented COVID‐19‐associated deaths among Veterans. The highest weekly counts for cases were observed in Wave 4 (Omicron); however, the highest weekly counts for known associated deaths were observed during Wave 2 (the first winter). See Figure 1.

### 4.3 Undocumented COVID‐19 Deaths

**Excess respiratory deaths:** Overall, we estimated 85,937 excess respiratory deaths within the Veteran population during the pandemic period inferring from documented influenza and pneumonia deaths.

**Excess all‐cause deaths:** The Farrington method [15, 16] was used to obtain the excess all‐cause deaths among enrolled Veterans, resulting in a total of 45,896 excess all‐cause deaths within Veterans—to be conservative, only deaths above the 95% upper bound were counted here.

**Inferred COVID‐19 deaths:** We obtained the weekly maximum of excess respiratory deaths and excess all‐cause deaths, which we subsequently used as ‘Inferred COVID‐19 Deaths’ to inform our case estimates. Overall, there were a total of 90,750 Inferred COVID‐19 deaths within Veterans, which resulted in a 3.72 ratio between inferred and documented COVID‐19 deaths.

**Figure 2:**
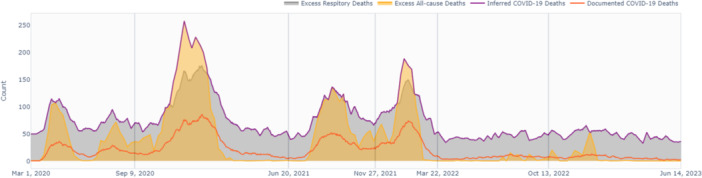
The daily counts for excess respiratory deaths (‘Gray’ filled line), excess all‐cause deaths (‘Yellow’ filled line), inferred COVID‐19 deaths (‘Purple’ line) and documented COVID‐19 deaths (‘Red’ line) among Veterans over the course of the pandemic.

Figure 2 shows the death counts over time for excess respiratory, excess all‐cause, and inferred COVID‐19 deaths.

### 4.4 IFR Variations over Time

**Constant IFR:** The initial IFR value of 65 per 10,000 is reported by Lu et al. [6] The latest COVID‐19 case and death counts from the CDC [14] is used to update the IFR value, which is 55 per 10,000.

**Wave‐based IFRs:** The same COVID‐19 case and death counts from CDC [14] were used to calculate individual IFR values for each wave (per 10,000 infections):

- Wave 1: 80
- Wave 2: 80
- Wave 3: 60
- Wave 4: 30
- Wave 5: 20
- Wave 6: 20

**Age‐adjusted Wave‐based IFRs**: We derived the IFR ratios for different age groups from the systematic analysis on COVID‐19 infection–fatality ratios presented by Sorensen et al. [17]. See Table 1. The age group based IFR ratios and the previously computed ‘Wave‐based IFR values’ were used to compute the age‐adjusted wave‐based IFR values as follows: *IFR_ Ratio*_*AgeGroup*_ * IFR_*wave*_. Resulting age‐adjusted IFR values for each wave are shown in Table 1.

**Table 1:** IFR ratios by age group derived based on systematic analysis of COVID‐19 infection‐fatality ratios17 are presented per 10,000 infections. ‘Overall IFR’ is considered to be 75.4 per 10,000.

Age group	IFR for age group†	IFR ratio for age group^‡^
< 20	0.5	0.006
20–29	2.9	0.039
30–39	10.3	0.137
40–49	28.0	0.371
50–59	62.6	0.83
60–69	169.2	2.244
70–79	478.9	6.532
80+	2,224.7	29.506

^†^ IFR for Age Group’ values are derived from Sorensen et al [17] per 10,000 infections.

^‡^ IFR Ratio for Age Group = IFR for Age Group/Overall IFR.

Using an initial IFR value of 65 per 10,000 reported by Lu et al. [6] to estimate COVID‐19 Veteran case counts, we estimated that there were 7,952,414 symptomatic COVID‐19 cases during the study period. The ratio of estimated to documented cases was 10.5. Additionally, we generated case estimates using the updated IFR value of 55 per 10,000. These results show 9,398,308 total estimated cases with a ratio of 12.35, which is more than the total Veteran population. Wave‐specific IFRs resulted in even higher estimates, with a calculated overall estimate of 12,764,936 cases and a ratio of 16.8 between estimated and documented cases. The age‐adjusted wave‐based IFRs (shown in Table 2) estimate COVID‐19 cases among Veterans was 4,168,754, corresponding to a ratio of 5.48.

**Table 2:** Wave‐based age‐adjusted IFR values computed based on wave‐based IFR values and IFR ratios for age groups (from Table 2) are presented per 10,000.

	< 20	20–29	30–39	40–49	50–59	60–69	70–79	80 +
**Wave 1**	0.5	3.1	10.9	29.7	66.4	179.5	508.1	2,360.5
**Wave 2**	0.5	3.1	10.9	29.7	66.4	179.5	508.1	2,360.5
**Wave 3**	0.4	2.3	8.2	22.3	49.8	134.7	381.1	1,770.4
**Wave 4**	0.2	1.2	4.1	11.1	24.9	67.3	190.6	885.2
**Wave 5**	0.1	0.8	2.7	7.4	16.6	44.9	127.0	590.1
**Wave 6**	0.1	0.8	2.7	7.4	16.6	44.9	127.0	590.1

**Figure 3:**
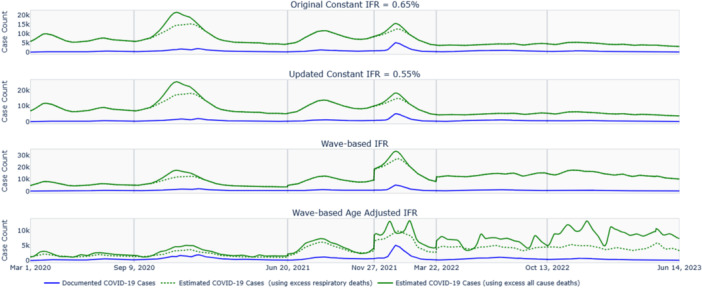
The estimated COVID‐19 cases (in ‘Green’ lines) and the documented COVID‐19 case counts (in ‘Blue’ lines) are shown for each of the IFR variation that were taken into consideration (original constant IFR, updated constant IFR, wave‐based IFR, and wave‐based age adjusted IFR). The solid ‘Green’ line indicates results when all‐cause deaths are considered, whereas the dotted ‘Green’ line displays estimates when the excess respiratory deaths are taken into account.

**Figure 4:**
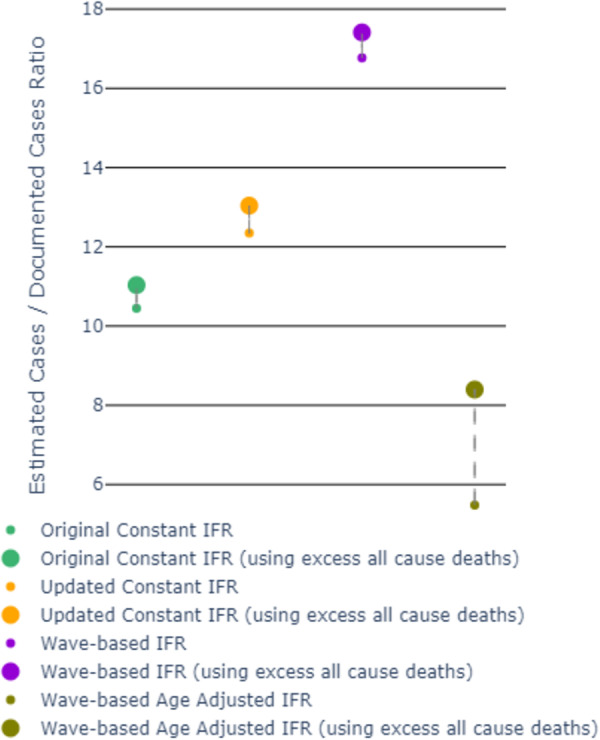
For each IFR variation that was taken into consideration (original constant IFR, updated constant IFR, wave‐based IFR, and wave‐based age adjusted IFR), the ratios between estimated and documented COVID‐19 cases are shown. Here, ratios for both when excess respiratory deaths and all‐cause deaths are taken into account are indicated.

Figure 3 displays the estimated COVID‐19 cases for each of the IFR variations mentioned above in addition to the cases that have been reported so far in the pandemic. It also illustrates the ratios between estimated and documented cases over time. For each IFR variation, the overall ratios between estimated and documented cases are shown in Figure 4. Next, we extended the mMAP adjusted version to include excess all‐cause deaths throughout the pandemic. Case estimations and their ratios when excess all‐cause deaths are considered are also shown in Figure 3 and Figure 4, respectively. As indicated by the results, extending the mMAP adjusted version to include excess all‐cause deaths along with age‐adjusted wave‐specific IFR values yielded a total of 6,387,975 estimated COVID‐19 cases for a ratio of 8.4. Estimated and documented case counts, along with their ratios, are presented by pandemic wave in Table S1 (see Supplementary Materials).

### 4.5 COVID‐19 Case Estimations by Race

**Figure 5:**
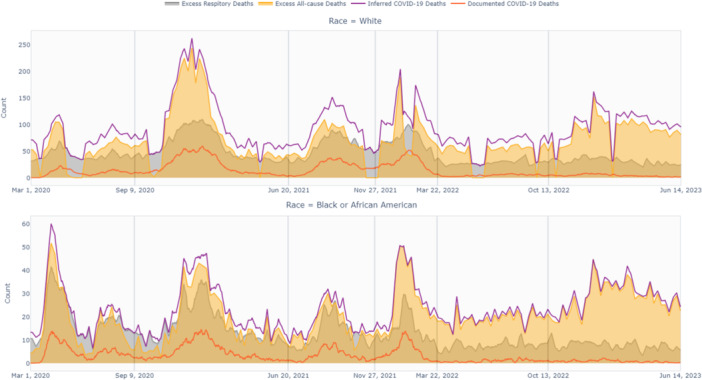
The daily counts for excess respiratory deaths (‘Gray’ filled line), excess all‐cause deaths (‘Yellow’ filled line), inferred COVID‐19 deaths (‘Purple’ line) and documented COVID‐19 deaths (‘Red’ line) among White and Black or African American Veteran groups over the course of the pandemic are shown.

Here, we present COVID‐19 case estimation results among different racial groups, with a focus on *White* and *Black or African American* Veterans. Figure 5 shows the death counts over time for excess respiratory, excess all‐cause, and inferred COVID‐19 deaths for both *White* and *Black or African American Veterans*. The results show that during the early stages of the pandemic, there was a high ratio of excess deaths to known deaths among *White* Veterans. Conversely, as the pandemic progressed, the ratio increased among *Black or African American* Veterans.

**Figure 6:**
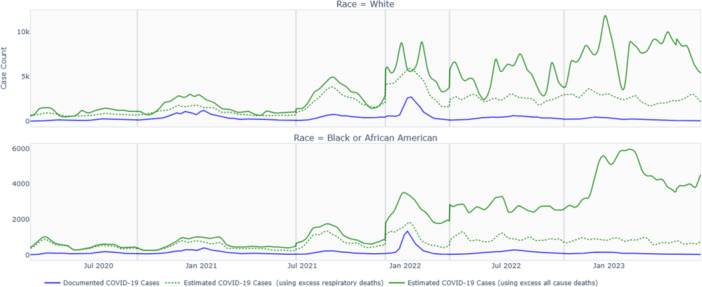
When wave‐based age adjusted IFR values are considered, the estimated COVID‐19 cases (in ‘Green’ lines) and the documented COVID‐19 case counts (in ‘Blue’ lines) for White and Black or African American demographic groups are shown. The solid ‘Green’ line indicates results when all‐cause deaths are considered, whereas the dotted ‘Green’ line displays estimates when the excess respiratory deaths are taken into account.

Figure 6 shows the estimated COVID‐19 cases for each racial group when the above inferred COVID‐19 deaths are used along with the wave‐based age adjusted IFR values (in Table 2). Figure S1 in the supplemental materials shows the same results as a percentage of the population. As indicated by the results, the eventual cumulative infection for *Black or African American* Veterans was much higher (exceeding their entire population) when compared to *White* Veterans.

## 5 Discussion

Our study highlights that the number of COVID‐19 infections inferred during the pandemic was at least several times greater than the documented cases over the entire study period, with the ratio between documented and expected infections varying over time and among different demographic groups. This marked underascertainment was significant, underscoring the limitations of relying solely on reported case numbers to understand the true extent of the pandemic. The variability in the ratio over time and across different populations points to inconsistencies in testing availability, public health reporting, and possibly the varying degrees of severity of illness in different groups.

Interestingly, the data revealed that all‐cause excess mortality closely matched excess respiratory mortality. We used a conservative estimate of all‐cause mortality that exceeded estimated respiratory deaths during large waves. Excess deaths attributable to COVID‐19, but not recorded as influenza or pneumonia deaths, likely occur at all times throughout the pandemic. This is consistent with non‐respiratory COVID‐19 deaths, potential deaths due to delay in care from an overwhelmed healthcare system, or respiratory COVID‐19 deaths occurring at home or at least outside of the VA healthcare system. However, estimates using excess all‐cause mortality later in the study period appear less reasonable, suggesting that excess all‐cause mortality may only be a surrogate for pandemic‐related mortality during the early period [18].

Additionally, excess deaths observed in the later period of the pandemic (2022–2023) may not solely reflect acute COVID‐19 mortality. Post‐acute sequelae of SARS‐CoV‐2 infection (PASC) has been associated with elevated mortality risk [18] but other causes of excess mortality likely become more important after the first year of COVID‐19. Attributing all excess deaths during this period to acute COVID‐19, and using them to back‐calculate infection rates, will therefore introduce some upward bias in our later estimates, especially after the initial impact period.

The ratio between excess deaths and documented COVID‐19 deaths shifted over time by race. Our analysis suggested an underestimate of deaths amongst *White* Veterans early in the pandemic, while later on there was an apparent underestimation amongst both *White* and *Black or African American* Veterans. This could be explained in part by greater access to healthcare outside of VA, for example, by *White* Veterans early in the pandemic. Other explanations include excess death not attributable to COVID‐19 and decreased ascertainment across groups.

By the end of the study period, our results suggested that the majority of Veterans had been infected. However, the time frame for different populations to approach near‐unity with respect to cumulative primary infection, meaning nearly everyone having been infected at least once, varied considerably. This variation could be attributed to differing levels of exposure, the implementation and adherence to public health measures, and differences in individual behavior and susceptibility. Our case estimates reflect first lifetime incident COVID‐19 infections and do not account for reinfection. As the pandemic progressed, reinfection became increasingly common, and later waves and changing mortality likely reflect that. Furthermore, as a largely immune population accumulated prior infection and vaccination, the IFR itself continues to shift, complicating assumptions that underpin our estimates. Accounting for reinfection could be particularly important when applying this framework to ongoing seasonal surveillance, where distinguishing between first infections and reinfections has meaningful implications for estimating true disease burden.

Our findings are consistent with previous studies, which estimated very large ratios between estimated and reported cases [6, 7, 9]. In the supplement, we also applied other algorithms to VA data but used assumptions that did not hold after the early period of the pandemic. They too showed a very large estimate of cases compared to documented cases. We did relax certain assumptions about mortality with respect to age (given VA's older age distribution) and wave, to enable inferences across the entire pandemic period. This tempered more naive estimates and showed reasonable alignment with seroprevalence data, which tracks the presence of virus‐specific antibodies in different age groups over time. Seroprevalence studies provide valuable cross‐sectional snapshots that, when complemented with our results, offer a more comprehensive picture of infection spread [19, 20]. For the purposes of analysis, we assumed that all differences by race were mediated through age. This is likely not true and is a limitation. Our analysis of demographic differences was limited to race and age, and did not incorporate broader social determinants of health such as income or geographic factors. Incorporating such variables could provide a more nuanced understanding of the differential ascertainment biases observed across demographic groups and further strengthen the implications of our findings for future pandemic preparedness. We acknowledge this as an area for future work.

This study demonstrates a magnitude of underascertainment bias that is likely much larger than most other measurement errors and biases documented during the course of the pandemic. These biases also change over time, presumably related to the expected value of information from testing at that time [21]. This suggests that efforts to pinpoint exact infection numbers may be misdirected. It also calls into question what forecasts mean. Data used to train models for forecasting are subject to bias over time that may vary by demographic group. They therefore capture transmission dynamics blurred by changing biases in the data collection system. Therefore, most forecasts will not predict future cases but future ascertained cases. Furthermore, it has been reported that forecasts do most poorly when dynamics shift (when we most need accurate forecasts) [22]. However, when applied to public health and healthcare system decision‐making, this suggests that the bias and quality of data when it is needed most may be when it is the worst. This affects the quality of decisions as well as which group may benefit or suffer the most. It also suggests that we need to understand the dynamics of ascertainment when making forecasts.

We relied heavily on published parameters to infer the total cumulative infections, which may be wrong for precisely the reasons outlined above. Therefore, these estimates should not be interpreted to be precise estimates but to help infer the scale of burden of disease and the amount of error in our measurements. To enhance understanding, comprehensive models that incorporate relevant real‐time data feeds could provide a more accurate ongoing picture of COVID‐19 impact on communities. Such integrated models could include data from hospitalizations, wastewater analyses, mobility patterns, and more real‐time reporting mechanisms, and thus aid in better decision‐making and resource allocation by offering more reliable and timely insights. While we refined IFR estimates by wave and age group, we did not explicitly incorporate vaccination status into our model. Because the IFR values used in this analysis were derived from CDC case and death counts, which reflects a mix of vaccinated and unvaccinated individuals, vaccine effectiveness is at least partially captured in our estimates. Nevertheless, more explicitly accounting for vaccination remains a limitation of our approach.

VA patients skew heavily male and have an age and disease distribution distinct from the overall population. They are part of the general population but there are likely special dynamics involved, and VA data will not capture the whole picture.

## 6 Conclusion

This comprehensive analysis underscores the inability of even large integrated healthcare systems to capture all COVID‐19 cases. We extended existing methods to account for variability in IFRs and broadened the scope to include all‐cause excess deaths. The discrepancies in case ascertainment rates, particularly across different demographic groups, highlight the need for more nuanced and adaptive approaches for data collection, data integration, and epidemiological estimation. Policymakers should be cautious of interpreting reported data as‐is, as it may under‐represent the true scale of the pandemic's impact on the Veteran population. Furthermore, it is important to recognize that most forecasting approaches predict what will be documented in the future, not what will happen in the future. More accurate estimation of infection rates is vital for developing effective health strategies and ensuring fair resource allocation, ultimately improving the health outcomes of Veterans.

## Author Contributions

Conceptualization: Wathsala N. Widanagamaachchi, Vanessa W. Stevens, and Makoto M. Jones. Data Curation: Wathsala N. Widanagamaachchi, Tina M. Willson, and Christian D. Dalton. Formal Analysis: Wathsala N. Widanagamaachchi and Makoto M. Jones. Funding acquisition: Matthew Samore and Makoto M. Jones. Methodology: Wathsala N. Widanagamaachchi and Makoto M. Jones. Supervision: Makoto M. Jones. Visualization: Wathsala N. Widanagamaachchi. Writing – original draft: Wathsala N. Widanagamaachchi and Makoto M. Jones. Writing – review and editing: Wathsala N. Widanagamaachchi, Tina M. Willson, Ariana R. Callahan, Bree Barbeau, Julia E. Lewis, Christian D. Dalton, Vanessa W. Stevens, Matthew Samore, Nathorn Chaiyakunapruk, and Makoto M. Jones.

## Ethics Statement

The study obtained ethics approval by the University of Utah and Veteran Affairs Institutional Review Boards (IRB_000146501).

## Conflicts of Interest

Some of the authors (MJ & MS) have received funding from the Centers for Disease Control and Prevention. The authors declare no other conflicts of interest.

## Supporting information

Supporting File

## Data Availability

The US Department of Veterans Affairs (VA) places restrictions on access to Veterans' healthcare data, including the de‐identified data set used in this study. These restrictions are due to the inclusion of report data and other details that make the data extremely difficult to fully de‐identify. VA has generally not allowed release of de‐identified data because they are usually re‐identifiable, at least in part. The data that support the findings of this study are available on request with an approved IRB protocol and VA Research and Development Committee Approval letter to the Veterans Information and Computing Infrastructure (VINCI@VA.gov).
