# Supplementary material for: How Many Veteran COVID‐19 Cases Were There during the Pandemic?
Source: J Med Virol. 2026 Jun 29;98(7):e71040. doi: 10.1002/jmv.71040 (PMC13311740; doi:10.1002/jmv.71040)
Supplement: Supplementary file 1 — Supporting File [file JMV-98-e71040-s001.docx]

**How Many Veteran COVID-19 Cases Were There During the Pandemic?**

Supplementary Materials

This serves as a supplementary document to further expand upon the results for estimating the incidence of symptomatic COVID-19 cases among Veterans in the United States across different demographic groups from the beginning of the pandemic to the end of 2022.

# **S.1 Methods**

Lu et al.^4^ introduced several complementary methods for estimating early cumulative incidence of symptomatic COVID-19 cases. In the main manuscript, we focused on the mMAP approach; in this supplement, we expand on the application of the Divergence and COVID Scaling approaches to estimating the COVID-19 cases within the U.S. Veteran population. As noted by Lu et al.^4^, these methods rely on reporting of influenza-like illness (ILI). However, ILI data became unreliable during the COVID-19 pandemic due to changes in healthcare-seeking behavior and influenza testing practices, introducing substantial reporting biases.^4^ Please see the original work for details about these methods.

**S.2 Results**

## **Divergence**

Lu et al.^4^ discussed that since Divergence methods are based on ILI signal, they are only accurate during the early stages of the COVID-19 pandemic when ILI reporting was operating as usual. For this reason, we have only included through the first or second waves of the pandemic in our results to demonstrate these models.

### *div-Baseline*

The first, and simplest Divergence method is *div-Baseline*, which uses a historical weekly average of past flu seasons to estimate COVID-19 cases. As shown in Figure S1, the estimates quickly fall into the negatives beginning in the first wave and remain in the negatives for nearly the entire study period, with a few exceptions. This is likely because once COVID-19 was recognized as its own syndrome and health-seeking behaviors changed, reported ILI cases fell far below the historical baseline.

Figure S1 div-Baseline predicted COVID-19 cases (green lines) compared to observed COVID-19 cases (blue line) in the U.S. Veteran population

### *div-Hist*

The *div-Hist* method is based on seasonal time series decomposition, fitted to observed 2019-2020 ILI. This model’s estimates also quickly fell into the negatives and then returned null values, as shown in Figure S2. This is likely due to the ILI signal largely disappearing several months into the pandemic.

Figure S2 div-Hist predicted COVID-19 cases (green lines) compared to observed COVID-19 cases (blue line) in the U.S. Veteran population.

*div-Vir*

The *div-Vir* method is based on observations of positive influenza test statistics. This model estimated an initially higher number of cases and generally tracked with waves but estimates fell below observed documented cases as the pandemic progressed, likely due to lack of influenza testing done during the pandemic. The predictions for the first wave of the pandemic are shown in Figure S3.

Figure S3 div-Vir predicted COVID-19 cases (green lines) compared to observed COVID-19 cases (blue line) in the U.S. Veteran population.

### *div-IDEA*

The *div-IDEA* method is an epidemiological model that accounts for control activities and behaviors. The assumptions made in this model hold for the early part of the pandemic but fail as behaviors change later in the pandemic. Eventually, we see the estimates fall below observed documented cases, especially during the fourth wave, similar to the *div-Vir* method. The predictions for the first wave are shown in Figure S4.

Figure S4 div-IDEA predicted COVID-19 cases (green lines) compared to observed COVID-19 cases (blue line) in the U.S. Veteran population.

## **COVID Scaling**

The COVID Scaling method uses ILI healthcare visits as well as COVID-19 test statistics to infer the proportion of ILI due to COVID in the full population. Similar to other models, we see the estimate fall below observed COVID-19 estimates after the early period of the pandemic as shown in Figure S5.

Figure S5 COVID Scaling predicted COVID-19 cases (green lines) compared to observed COVID-19 cases (blue line) in the U.S. Veteran population.

**S.3 Estimated and Documented Cases by Wave**

To provide a more granular view of the discrepancy between documented and estimated cases, Table S1 presents the estimated and documented COVID-19 case counts alongside their ratios for each pandemic wave. These estimates were derived from the mMap model incorporating excess all-cause death and wave- and age-adjusted IFRs.

Table S1. Estimated and Documented Veteran COVID-19 Cases by wave.

| **Wave** | **Period** | **Documented Cases** | **Estimated Cases*** | **Ratio (Estimated:Documented)** |
| --- | --- | --- | --- | --- |
| 1 | Mar 1, 2020 – Sep 8, 2020 | 50,820 | 379,982 | 7.48 |
| 2 | Sep 9, 2020 – Jun 19, 2021 | 197,311 | 713,014 | 3.61 |
| 3 | Jun 20, 2021 – Nov 26, 2021 | 104,861 | 725,356 | 6.92 |
| 4 | Nov 27, 2021 – Mar 21, 2022 | 193,754 | 987,087 | 5.09 |
| 5 | Mar 22, 2022 – Oct 12, 2022 | 677,222 | 1,320,727 | 10.112 |
| 6 | Oct 13, 2022 – Jun 14, 2023 | 83,512 | 2,261,809 | 27.08 |
| Overall | Mar 1, 202 – Jun 14, 2023 | 760,734 | 6,387,975 | 8.40 |

* Estimated using mMAP model incorporating excess all-cause deaths and wave and age adjusted IFRs.
